# Uveitis Associated with Conventional Cigarette Smoking and E-Cigarette Use: A Narrative Review

**DOI:** 10.3390/medicina62061198

**Published:** 2026-06-22

**Authors:** Alan Y. Hsu, Chun-Ju Lin, Ning-Yi Hsia, Yi-Ching Shao, Chun-Chi Chiang, Chun-Ting Lai, Chia-Yun Chen, Yi-Yu Tsai

**Affiliations:** 1 Department of Ophthalmology, China Medical University Hospital, China Medical University, Taichung 404327, Taiwan; alanhsu1221@gmail.com (A.Y.H.);; 2 School of Medicine, College of Medicine, China Medical University, Taichung 404327, Taiwan; 3 Department of Optometry, Asia University, Taichung 404327, Taiwan; 4 Graduate Institute of Biomedical Sciences, College of Medicine, China Medical University, Taichung 404327, Taiwan

**Keywords:** vaping, e-cigarette, cigarette, uveitis, review

## Abstract

*Background and Objectives*: Uveitis is an inflammatory condition of the uveal tract and has been associated with cigarette smoking. However, the relationship between uveitis and electronic cigarette (e-cigarette) use remains poorly understood. This review aims to give a brief overview of the current evidence regarding the association between uveitis and both conventional cigarette smoking and e-cigarette use. *Materials and Methods*: A narrative literature review was conducted using PubMed and Scopus databases. Relevant peer-reviewed publications were identified using the search terms “vaping,” “cigarette,” and “uveitis.” *Results*: Multiple studies have demonstrated a significant association between cigarette smoking and an increased risk of uveitis. In contrast, evidence examining the relationship between e-cigarette use and uveitis is limited. *Conclusions*: Uveitis is a potentially vision-threatening ophthalmologic condition, and improved understanding of its risk factors is essential for optimal patient care. While the association between conventional cigarette smoking and uveitis is well established, current evidence regarding e-cigarette use remains insufficient. Further research is needed to clarify the potential role of e-cigarette use in the development of uveitis.

## 1. Introduction

Uveitis, an inflammatory condition affecting the uveal tract of the eye, is a significant cause of visual impairment among working-age adults in the United States [1]. The uveal tract, which consists of the iris, ciliary body, and choroid, plays a critical role in maintaining ocular function through regulation of blood supply and intraocular homeostasis. Inflammation of these structures can therefore have significant consequences, ranging from mild visual disturbances to severe and permanent vision loss if not promptly recognized and treated. Clinically, uveitis is a heterogeneous condition that can be broadly divided into infectious and noninfectious types, depending on the underlying etiology. Infectious uveitis is caused by microbial pathogens such as bacteria, viruses, fungi, or parasites, whereas noninfectious uveitis is typically immune-mediated and often associated with systemic inflammatory or autoimmune diseases [2].

Although the exact pathogenesis of noninfectious uveitis is not fully understood, inflammation is widely recognised as the central underlying mechanism [3]. Furthermore, numerous studies have demonstrated a clear association between uveitis and conventional cigarette smoking, with inflammation again proposed as the key underlying mechanism linking the two [4]. However, the relationship between a history of e-cigarette use and uveitis risk remains less well established. E-cigarettes, also known as vaping devices, have gained widespread popularity over the past decade, particularly among adolescents and young adults. These devices function by heating a liquid solution that typically contains nicotine, propylene glycol, glycerin, and various flavoring agents to produce an inhalable aerosol. While often marketed as a safer alternative to conventional cigarettes, emerging evidence has also suggested that e-cigarette aerosols also contain potentially harmful substances capable of inducing oxidative stress and inflammatory responses, and potentially leading to increased risk for uveitis as well. However, current evidence remains limited in this regard. Accordingly, this narrative review aims to summarise the current evidence regarding the association between a history of conventional cigarette use as well as a history of e-cigarette use and uveitis.

## 2. Materials and Methods

A non-systematic literature search of the PubMed and COCHRANE electronic databases was conducted to identify studies examining the association between the history of conventional cigarettes or e-cigarette usage and the risk of uveitis. The search covered publications from January 2010 to December 2026. The search strategy employed a combination of keywords to encompass all relevant terminology for conventional cigarette or e-cigarette exposure and ocular inflammatory outcomes. Keywords included “traditional cigarette,” “e-cigarette,” “vaping,” “uveitis,” and “intra-ocular inflammation.” Additionally, only human studies published in English.

Studies that did not clearly address the association between a history of either the use of conventional cigarettes or e-cigarettes and ocular inflammation were excluded. A total of seven studies were included in this review.

## 3. Results

### 3.1. Role of Cigarettes in Developing Uveitis

Smoking is an increasingly common lifestyle habit that has been strongly associated with numerous health complications, including cancer, heart disease, and lung disease. Cigarette smoke contains harmful compounds such as nicotine, tar, carbon monoxide, and nitrogen oxides, all of which have detrimental effects on the body [5]. These substances have been shown to promote the generation of free radicals, leading to cellular damage and activation of oxidative stress–sensitive signalling pathways, thereby further exacerbating tissue injury [6]. Such an effect could contribute to an overall increased systemic inflammatory state, placing smokers at greater risk for inflammation-associated conditions. One such condition is uveitis. Uveitis is characterized by increased intraocular inflammation and has previously been associated with smoking. Several studies have evaluated the relationship between a history of conventional cigarette activity and uveitis, as summarised in Table 1.

In the retrospective study by Yuen et al. [7], this population-based case–control study included 100 patients with confirmed uveitis and 528 randomly selected controls from the study population. Yuen et al. found that patients with a history of conventional cigarette use had 2.33 times increased risk for developing uveitis compared with those who never had a history of smoking (95% CI: 1.22–4.45).

Roesel et al. also recruited 115 patients with a history of smoking and found increased risk for uveitis (OR 1.8, CI: 1.2–2.9, *p* = 0.007) [8].

In a third study, Gonzalez et al. recruited 5106 participants and, through multivariate analysis, found that a history of conventional cigarette use was associated with an increased odds ratio for uveitis (OR: 3.18, CI: 1.59–6.37, *p* = 0.003) after adjusting for age and gender [9].

Finally, in a retrospective study by Lin et al., after retrospectively recruiting 564 patients with a history of ocular inflammation, they found that those with a history of conventional cigarette use were at an increased risk for ocular inflammation compared to those who had never smoked (OR: 2.2, CI: 1.7–3.0, *p* < 0.001) [10].

Taken together, these studies demonstrated that history of conventional cigarette smoking is associated with an increased risk of ocular inflammation.

### 3.2. E-Cigarette and Association with Uveitis

E-cigarettes, or vaping devices, as mentioned earlier, are a relatively recent phenomenon that aerosolize chemical substances and have seen a marked increase in use since their introduction around 2010. E-cigarettes heat a liquid mixture that typically contains nicotine and additives such as propylene glycol, glycerin (glycerol), and various flavouring agents. Heating these substances can generate carbonyl compounds and induce oxidative stress, which may contribute to an overall increase in systemic inflammation [11]. Stress responses triggered by smoking can also involve multiple immunological pathways. These include enhanced neutrophil activation, increased macrophage activity, and a shift toward pro-inflammatory T-helper immune responses. In addition, smoking may impair regulatory T-cell (Treg) function, reducing the body’s ability to suppress inappropriate immune and inflammatory activity. Together, these effects can promote immune dysregulation and increase the risk of developing ocular inflammatory conditions [12].

Both conventional cigarettes and e-cigarettes can impair vascular health, largely due to nicotine, a potent vasoconstrictor that reduces blood flow and promotes endothelial dysfunction. In the eye, disruption of the blood–aqueous and blood–retinal barriers is a key step in the development of uveitis. Endothelial dysfunction can increase vascular permeability, allowing inflammatory mediators and immune cells to infiltrate ocular tissues. Chronic exposure to nicotine and other toxic compounds may therefore predispose individuals to recurrent or more severe episodes of uveitis [12]. Furthermore, because e-cigarettes contain similar components—particularly nicotine—it is plausible that the same inflammatory pathways triggered by conventional cigarette smoking may also be activated in individuals with a history of e-cigarette use.

**Figure 1:**
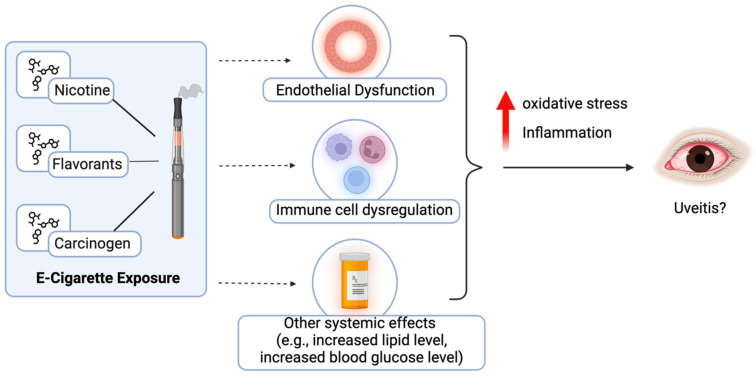
E-cigarette composition and its potential links with inflammation.

Uveitis, as mentioned earlier, is an ocular inflammatory condition characterised by inflammation of the uveal tract and is theoretically associated with e-cigarette use. Conventional cigarette smoking—and by extension, e-cigarette use—is strongly linked to systemic inflammation, providing a plausible biological pathway connecting these exposures to the development of uveitis (refer to Figure 1).

This persistent pro-inflammatory state can disrupt normal immune regulation and contribute to endothelial dysfunction, ultimately compromising the integrity of the blood–ocular barrier. Once this barrier is impaired, immune cells and inflammatory mediators can infiltrate intraocular tissues, triggering the characteristic inflammation seen in uveitis.

However, studies directly examining the relationship between history of e-cigarette use and uveitis remain limited. As part of this review, we also attempted to summarise the available studies and case reports that have described a potential association to date (refer to Table 2). It should be emphasised that these studies provide only indirect evidence of an association between e-cigarette use and the risk of uveitis. Among the three studies included, only one was a large-scale multicenter retrospective study, which provides comparatively stronger evidence than the other two publications included in this narrative review. In addition, one study by Nguyen et al. evaluated ocular symptoms rather than confirmed cases of Uveitis, limiting the extent to which its findings can be interpreted as evidence of ocular inflammation associated with e-cigarette use. Nevertheless, given the limited number of studies currently available examining the association between e-cigarettes and uveitis, the reports by Nguyen et al. and Iqbal et al. were also included in this narrative review to provide a broader perspective and preliminary insight into this potential association.

Among the three publications included in this narrative review pertaining to e-cigarettes, the study by Hsu et al. is the only large-scale investigation examining the association between a history of e-cigarette use and uveitis. In a study by Hsu et al. [3], a multicenter electronic health records registry was used to assess the risk of uveitis among individuals with and without a history of e-cigarette use. The authors employed propensity score matching to account for potential confounding factors such as age, sex, and comorbid conditions. This methodological approach strengthens the validity of their findings by attempting to minimize baseline differences between comparison groups. Hsu et al. reported an increased risk of uveitis among e-cigarette users compared with users with no history of e-cigarette use (hazard ratio (HR): 2.58), suggesting more than a twofold increase in risk. Hsu et al. also noted that e-cigarette users were at increased risk for iridocyclitis and choroioretinal inflammation subtype of uveitis. Notably, the study also demonstrated an elevated risk of uveitis among conventional cigarette smokers compared with non-smokers (HR: 1.28), reinforcing previously established associations between smoking and ocular inflammation.

Importantly, individuals with a dual history of e-cigarette and conventional cigarette use had a higher risk of uveitis compared with those with no history of e-cigarette use (HR: 1.35) and compared with non-users overall (HR: 1.84). Additionally, dual users exhibited a higher risk than individuals who used only conventional cigarettes (HR: 1.39). These findings raise the possibility of additive or synergistic effects between combustible tobacco and e-cigarette exposure. One potential explanation is that dual users may experience cumulative exposure to a broader spectrum of toxicants, including both combustion-derived products and aerosolized chemicals from e-cigarette devices. This combined exposure may amplify oxidative stress and inflammatory responses, thereby increasing susceptibility to intraocular inflammation.

While the study by Hsu et al. provides valuable epidemiological insight, it is important to consider its limitations. The reliance on diagnostic coding introduces the possibility of misclassification bias, particularly given the absence of standardized codes specifically for e-cigarette use. Furthermore, the observational nature of the study precludes definitive conclusions regarding causality. Nonetheless, the findings offer important preliminary evidence supporting a potential association between e-cigarette use and uveitis.

In addition to large-scale database studies, individual case reports can also provide further evidence of potential links between uveitis and e-cigarette use. In a case report by Iqbal et al., a 41-year-old male with no significant past medical history developed panuveitis following a history of daily usage of e-cigarettes for two years [13]. However, as this is a single case report describing uveitis in an indivudal with history of e-cigarette use, it is inherently subject to a high risk of reporting bias and should therefore be interpreted with caution. Nevertheless, given the limited number of published case reports examining uveitis following e-cigarette use, this case report was included in the present narrative review.

Lastly, this narrative review also included a cross-sectional study that may suggest a potential association between a history of e-cigarette use and an increased risk of ocular inflammation. However, it should be emphasized that this study did not recruit patients with confirmed cases of uveitis. Instead, it indirectly evaluated nonspecific ocular symptoms reported after a history of cigarette and e-cigarette use, which limits the ability to draw definitive conclusions regarding the relationship between a history of e-cigarette use and ocular inflammatory disease. However, given the limited number of publications on this topic, this study by Nguyen et al. may still offer a meaningful perspective to the existing literature. In this study, Nguyen et al. employed a cross-sectional design and recruited 4351 individuals to assess ocular symptoms occurring after a history of conventional cigarette as well as e-cigarette use. Although this study could not directly evaluate an association with uveitis, the ocular symptoms assessed—including ocular discomfort, pain, redness, blurred vision, and light sensitivity—are commonly associated with uveitis and may indirectly reflect its manifestations [14]. Nguyen et al. found that participants who used conventional cigarettes had a higher likelihood of experiencing severe ocular burning or stinging and blurred vision compared with all other participants. These findings are consistent with the known inflammatory effects of conventional cigarette smoke on ocular tissues. Additionally, individuals with a lifetime history of both e-cigarette and conventional cigarette use (dual users) were more likely to report blurred vision and foreign body sensation than participants in other exposure groups. This observation further supports the notion that combined exposure may exacerbate ocular surface inflammation and, possibly, intraocular inflammation. While these symptoms are nonspecific and cannot be equated directly with uveitis, their increased prevalence among users suggests that conventional cigarette and e-cigarette exposure may contribute to a pro-inflammatory ocular environment. It is also important to recognize the limitations inherent in cross-sectional studies. Because exposure and outcomes are assessed simultaneously, it is not possible to determine temporal relationships or infer causality. Additionally, reliance on self-reported symptoms introduces potential recall bias, selection bias and subjective variability. Despite these limitations, the study provides useful insight into the broader spectrum of ocular symptoms associated with tobacco and e-cigarette use and highlights the need for more targeted research on intraocular inflammation. However, future prospective studies may be required to mitigate such bias and provide further evidence toward the association between uveitis and e-cigarette use.

**Table 2:** Studies and reports relevant to the possible association between e-cigarette use, ocular symptoms, and uveitis.

Citation	Year of Publication	Type of Study	Number of Patients	Findings
Hsu et al. [3]	2025	Retrospective	419,325	Patients with history of e-cigarette use are at an increased risk for uveitis compared to control
Nguyen [14]	2023	Cross-sectional	4351	Higher likelihood of ocular symptoms from among those with history of combined e-cigarette and cigarette use
Iqbal et al. [13]	2022	Case report	1	Pan-uveitis reported in a case with e-cigarette history

Taken together, the available evidence suggests a potential association between e-cigarette use and uveitis, although current data remain sparse and heterogeneous. The relative consistency of findings across various study designs—including retrospective analyses, case reports, and cross-sectional surveys—potentially supports the hypothesis that e-cigarette use may contribute to ocular inflammation. However, the absence of prospective studies and the lack of standardized measures of exposure limit the strength and generalizability of these conclusions.

## 4. Future Directions

### 4.1. Need for Robust Epidemiological Evidence

To further clarify the relationship between the history of conventional cigarette use, e-cigarette use, and uveitis, additional research is required, as current evidence remains limited and largely observational. Although early studies suggest that either a history of conventional cigarette smoking or e-cigarette use may be associated with an increased risk of ocular inflammation, definitive conclusions cannot yet be drawn because of methodological limitations, small sample sizes, and the lack of long-term follow-up data. One study reported an increased risk of both anterior and posterior uveitis among individuals with a history of e-cigarette use, while a separate case report described panuveitis in an e-cigarette user [3,13]. With the global rise in the use of e-cigarettes—particularly among younger populations—it is increasingly important to develop a deeper understanding of how these exposures influence the onset and progression of inflammatory eye diseases such as uveitis. A key priority for future research is the implementation of well-designed longitudinal cohort studies. By following individuals over extended periods, prospective studies can help establish temporal relationships between exposure and disease development, thereby providing stronger evidence for causality than retrospective or cross-sectional designs.

Such studies should include large and diverse populations to enhance generalizability and account for potential confounding factors, including socioeconomic status, underlying health conditions, and environmental exposures. In addition, repeated follow-up assessments would allow researchers to evaluate disease recurrence, severity, and treatment outcomes in relation to continued or discontinued exposure.

Beyond epidemiological approaches, laboratory and animal studies are also essential for clarifying the biological mechanisms linking conventional cigarette and e-cigarette use to uveitis. Emerging evidence suggests that both conventional cigarette smoke and e-cigarette aerosols can activate pro-inflammatory pathways. Taken together, these findings support the hypothesis that a history of conventional smoking use may contribute to ocular inflammation through both systemic and local immune-mediated mechanisms. However, most existing laboratory studies have primarily focused on conventional cigarette smoke exposure rather than e-cigarette aerosols. Nevertheless, because conventional cigarette smoke and e-cigarette vapours share several chemical constituents, including nicotine, reactive carbonyl compounds, and particulate matter, it is biologically plausible that they may induce similar inflammatory and oxidative stress pathways. As such, the pathogenic effects observed with conventional cigarette smoking use may also extend, at least in part, to e-cigarette use, although this relationship requires direct experimental validation. Future studies that directly demonstrate these effects in ocular tissues while simultaneously distinguishing ocular-specific from systemic inflammatory mechanisms within a single, integrated experimental framework would substantially strengthen the biological plausibility of a causal relationship between conventional and e-cigarette use and uveitis. At present, many of the proposed pathophysiological mechanisms are inferred from separate laboratory and animal studies rather than from unified investigations specifically focused on ocular inflammatory disease.

### 4.2. Improved Exposure Assessment

A major limitation in current research is the lack of detailed and standardised exposure assessment. Behaviours related to conventional cigarette and e-cigarette use can vary widely in frequency, duration, and intensity, all of which may influence disease risk. For conventional cigarettes, pack-years remain a useful metric for estimating cumulative exposure and assessing the risk of outcomes such as uveitis. In contrast, measuring exposure in e-cigarette users is far more complex. Factors such as nicotine concentration, device type, voltage or temperature settings, puffing patterns, and e-liquid composition—including flavouring agents—can all affect the dosage of potentially harmful compounds produced from the e-cigarette device. Newer generations of e-cigarettes deliver nicotine and other chemical components differently from older devices, and variability across brands and formulations further complicates direct comparisons of future uveitis risk. User behaviours, such as chain e-cigarette use or alternating between devices, may also increase exposure in ways that are difficult to capture in retrospective studies. Future research should address this heterogeneity by standardising key variables, incorporating objective measures of exposure—such as nicotine levels—and utilising real-time monitoring tools to more accurately quantify patterns of use. Implementing these strategies will improve reproducibility and enable more reliable assessments of the relationships among dual use, cumulative exposure, and ocular outcomes such as uveitis.

### 4.3. Dual Use and Cumulative Risk

Another emerging concern is dual use, defined as the concurrent use of conventional cigarettes and e-cigarettes. Individuals who engage in dual use may be exposed to a broader and more complex array of toxic compounds, potentially producing additive—or even synergistic—effects on inflammatory pathways. Preliminary evidence, including findings reported by Hsu et al., suggests that dual users may have an elevated risk of uveitis. This heightened risk may be driven by cumulative oxidative stress, increased overall nicotine exposure, and overlapping activation of inflammatory mechanisms. Moreover, dual use may complicate the assessment of dose–response relationships, as varying frequencies of each product could differentially influence systemic and ocular inflammation. Future studies, involving longitudinal research incorporating multimodal imaging and survey methods, are still needed to better understand the effects.

### 4.4. Public Health and Preventive Strategies

Lastly, public health initiatives play a critical role in addressing the potential ocular risks associated with a history of conventional cigarette and e-cigarette use. The growing popularity of e-cigarettes—particularly among adolescents and young adults—raises concerns about long-term health consequences that are not yet fully understood. There is a widespread perception that e-cigarette use is a safer alternative to conventional cigarette smoking; however, emerging evidence suggests that e-cigarette aerosols contain numerous substances capable of inducing oxidative stress and inflammation. Public health campaigns should aim to correct misconceptions and raise awareness about potential ocular and systemic risks secondary to both conventional cigarettes and e-cigarette use. Targeted educational efforts could include school-based programs, social media outreach, and community engagement to emphasise the risks of exposure to either conventional cigarette and e-cigarettes. Furthermore, potentially integrating ophthalmologic screening into broader tobacco cessation initiatives may help identify early signs of inflammation or ocular damage, facilitating timely intervention. Policymakers and healthcare providers should collaborate to implement evidence-based regulations, such as restrictions on youth access, product labelling, and public messaging that highlights both short- and long-term health implications. Through these combined strategies, public health efforts can better mitigate the potentially serious ocular consequences associated with conventional cigarette and e-cigarette behaviours.

## 5. Conclusions

In conclusion, this narrative review summarizes the current evidence on the association between a history of cigarette and e-cigarette use and the risk of uveitis, while also highlighting important unanswered questions that warrant further investigation. Despite these gaps, the available evidence underscores the need for clinicians and researchers to remain cautious and attentive to the potential ocular risks associated with e-cigarette use. As e-cigarette use continues to rise in popularity worldwide—particularly among younger populations—a better understanding of its long-term health effects, including its impact on ocular health, is an increasingly urgent task for future researchers and clinicians.

## Figures and Tables

**Table 1:** A list of studies that explored the association between conventional cigarette use and uveitis.

Citation	Year of Publication	Type of Study	Number of Patients	Findings
Yuen et al. [7]	2015	Retrospective	100 patients with uveitis	Smoking history was associated with increased risk for uveitis (HR: 2.33, CI: 1.22–4.45, *p* < 0.01)
Roesel et al. [8]	2011	Retrospective	350 patients with uveitis	Increased risk of uveitis from patients with smoking history (OR 1.8, CI: 1.2–2.9, *p* = 0.007)
Gonzalez et al. [9]	2018	Retrospective	27 patients with uveitis	Patients with smoking history are at increased risk for uveitis (OR: 3.18, CI: 1.59–6.37, *p* = 0.003)
Lin et al. [10]	2010	Retrospective	564 patients with uveitis	Smoking history was associated with increased risk for uveitis (OR: 2.2, CI: 1.7–3.0, *p* < 0.001)

## Data Availability

All data pertinent to this manuscript have already been included. For further details, please contact the corresponding authors.
